# Serrated adenocarcinoma morphology in colorectal mucinous adenocarcinoma is associated with improved patient survival

**DOI:** 10.18632/oncotarget.16815

**Published:** 2017-04-04

**Authors:** Chung-Ta Lee, Yu-Chuan Huang, Liang-Yi Hung, Nan-Haw Chow, Pei-Fang Su, Chung-Liang Ho, Hung-Wen Tsai, Yi-Lin Chen, Shao-Chieh Lin, Bo-Wen Lin, Peng-Chan Lin, Jenq-Chang Lee

**Affiliations:** ^1^ Department of Pathology, National Cheng Kung University Hospital, College of Medicine, National Cheng Kung University, Tainan 70403, Taiwan; ^2^ Institute of Bioinformatics and Biosignal Transduction, College of Bioscience and Biotechnology, National Cheng Kung University, Tainan 70101, Taiwan; ^3^ Department of Biotechnology and Bioindustry Sciences, College of Bioscience and Biotechnology, National Cheng Kung University, Tainan 70101, Taiwan; ^4^ Department of Statistics, College of Management, National Cheng Kung University, Tainan 70101, Taiwan; ^5^ Department of Surgery, National Cheng Kung University Hospital, College of Medicine, National Cheng Kung University, Tainan 70403, Taiwan; ^6^ Department of Internal Medicine, National Cheng Kung University Hospital, College of Medicine, National Cheng Kung University, Tainan 70403, Taiwan

**Keywords:** colorectal cancer, mucinous adenocarcinoma, serrated adenocarcinoma, CIMP, BRAF

## Abstract

Colorectal mucinous adenocarcinoma (MAC) and serrated adenocarcinoma (SAC) share many characteristics, including right-side colon location, frequent mucin production, and various molecular features. This study examined the frequency of SAC morphology in MACs. We assessed the correlation of SAC morphology with clinicopathological parameters, molecular characteristics, and patient prognosis. Eighty-eight colorectal MACs were collected and reviewed for SAC morphology according to Makinen's criteria. We sequenced *KRAS* and *BRAF*, assessed CpG island methylator phenotype (CIMP) frequency, and analyzed DNA mismatch repair enzyme levels using immunohistochemistry in tumor samples. SAC morphology was observed in 38% of MACs, and was associated with proximal location (*P*=0.001), *BRAF* mutation (*P*=0.042), CIMP-positive status (*P*=0.023), and contiguous traditional serrated adenoma (*P*=0.019). Multivariate analysis revealed that MACs without both SAC morphology and CIMP-positive status exhibited 3.955 times greater risk of cancer relapse than MACs having both characteristics or either one (*P*=0.035). Our results show that two MAC groups with distinct features can be identified using Makinen's criteria, and suggest a favorable prognostic role for the serrated neoplastic pathway in colorectal MAC.

## INTRODUCTION

Mucinous adenocarcinoma (MAC) of the colorectum accounts for approximately 10% of colorectal cancers (CRCs) and is diagnosed when >50% of the tumor is composed of extracellular mucin pools containing malignant epithelia [1, 2]. MAC is more common in women; it presents in the right colon and is generally detected at more advanced stages than non-MAC colorectal cancers [3]. The prognostic significance of colorectal MAC is controversial [2–5], but a recent meta-analysis associated MACs with slightly poorer prognosis [3]. MACs exhibit a higher frequency of DNA mismatch repair (MMR) defects, microsatellite instability (MSI) and RAS/RAF/MAPK pathway mutations than non-MACs, and a lower incidence of p53 mutations [6–9]. MACs also more frequently exhibit a CpG island methylator phenotype (CIMP). This is characterized by CpG island hypermethylation in the promoter regions of carcinogenesis-related genes, resulting in epigenetic silencing [6, 8, 10, 11].

Serrated adenocarcinoma (SAC) of the colorectum was first described by Jass in 1992 as having a close structural and histochemical resemblance to hyperplastic polyps with glandular serration [12], and was defined as a CRC subtype in the 2010 WHO classification [1]. SAC is associated with malignant transformation of serrated polyps, including sessile serrated adenomas and traditional serrated adenomas, which constitute the so-called “serrated neoplastic pathway” [13–16]. Makinen, *et al*. refined the SAC histological criteria to include epithelial serrations, clear or eosinophilic cytoplasm, abundant cytoplasm, vesicular nuclei, absence of or <10% necrosis of the total surface area, mucin production, and cell balls and papillary rods in the mucin [15, 17]. A previous study using these criteria found that SACs constituted 9.1% of the CRC cohort (n=927), and approximately half of these SACs contained a residual serrated adenoma precursor [18]. SACs are more often located in the proximal colon than conventional adenocarcinomas, and are associated with a lower 5-year survival [18, 19].

SAC histological features have been validated by mRNA expression profiling, which demonstrated that SACs differ from conventional adenocarcinomas both morphologically and molecularly [20]. In contrast with conventional colorectal adenocarcinoma resulting from a conventional adenoma-carcinoma sequence, SACs have a higher frequency of BRAF or KRAS mutations and higher levels of CIMP [15, 21–23]. The cause or mechanism of CIMP in CRC is not yet known. BRAF mutation might be an early event in CIMP tumors [24] and might direct CIMP development [25].

CIMP and BRAF mutations are associated with serrated CRC development, and these mutations occur frequently in MACs and SACs [7, 8, 10, 11, 15, 21–23, 26]. MACs and SACs also share other features, including right-side colon location and frequent mucin production. Whether MACs with and without SAC morphology represent two distinguishable CRC types with different behaviors is unknown. This study examined the occurrence of SAC in MACs, the correlation with clinicopathological parameters and molecular characteristics, and the prognostic implications.

## RESULTS

### Clinicopathological characteristics

Colorectal MACs from 88 patients (50 men and 38 women) were included in our study. Mean patient age at surgery was 66 years. Tumors were located as follows: 10 (11%) in the cecum, 21 (24%) in the ascending colon, 12 (14%) in the transverse colon, 12 (14%) in the descending colon, 19 (21%) in the sigmoid colon, and 14 (16%) in the rectum.

### Diagnostic concordance between pathologists for SAC morphology evaluation

**Figure 1:**
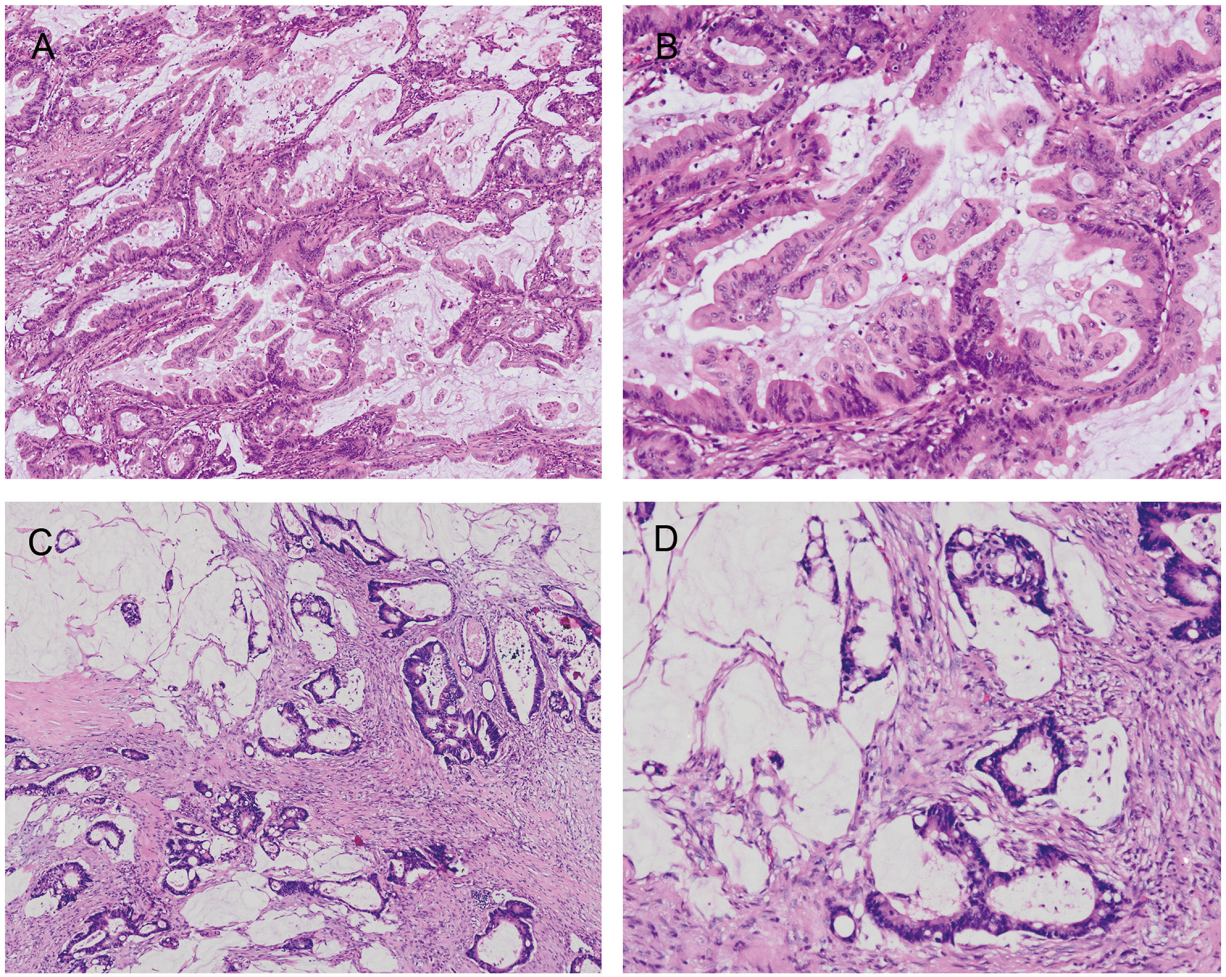
Representative MACs with and without SAC morphology MACs with SAC morphology **(A & B)** show prominent epithelial serration and typical cytology with eosinophilic cytoplasms and vesicular nuclei, in contrast with MACs without SAC morphology **(C & D)**.

There was 100% agreement among all three diagnosing pathologists in 61/88 cases (69%) separating MACs with or without SAC morphology (Figure 1). Inter-observer variation between the first investigator (Lee C. T.) and the two other observers showed moderate agreement (mean κ=0.578; range 0.462–0.693). The mean κ value for all pathologists was 0.579 (*P*<0.001), demonstrating moderate agreement between their diagnoses. Four cases without consensus were excluded from the SAC morphology analysis.

### SAC morphology correlation with clinicopathological and molecular characteristics

Thirty-two (38%) of the 84 cases that reached a consensus were diagnosed as MAC with SAC morphology (Table 1). MACs with SAC morphology were associated with proximal location (*P*=0.001), BRAF mutation (*P*=0.042), and CIMP-positive status (*P*=0.023) (Table 1).

**Table 1:** Clinicopathologic and molecular characteristics in 84 colorectal MACs with and without SAC morphology

Characteristic	MAC withoutSAC morphology(n=52)^a^	MAC with SACmorphology(n=32)^b^	*P*-value
Gender			0.255
Male	31 (59.6)	15 (46.9)	
Female	21 (40.4)	17 (53.1)	
Age			0.157
≤ 70 years	31 (59.6)	14 (43.8)	
> 70 years	21 (40.4)	18 (56.3)	
Location			0.001
Proximal colon	18 (34.6)	23 (71.9)	
Distal colon or rectum	34 (65.4)	9 (28.1)	
Differentiation			0.952
Well	9 (17.3)	5 (15.6)	
Moderate	36 (69.2)	22 (68.8)	
Poor	7 (13.5)	5 (15.6)	
AJCC TNM stage			0.889
Stage I	2 (3.8)	1 (3.1)	
Stage II	22 (42.3)	14 (43.8)	
Stage III	21 (40.4)	11 (34.4)	
Stage IV	7 (13.5)	6 (18.8)	
KRAS mutation (n=75)	19 (42.2)	13 (43.3)	0.924
BRAF mutation (n=73)	3 (7)	8 (26.7)	0.042
CIMP positive (n=77)	3 (6.5)	8 (25.8)	0.023
Defective mismatch repair protein	12 (23.1)	7 (21.9)	0.898

AJCC, American Joint Committee on Cancer; CIMP, CpG island methylator phenotype; MAC, mucinous adenocarcinoma; SAC, serrated adenocarcinoma.

^a^ Values are calculated for 52 MACs without SAC morphology unless specified otherwise, and are presented as number and percentage of patients.

^b^ Values are calculated for 32 MACs with SAC morphology unless specified otherwise, and are presented as number and percentage of patients.

**Figure 2:**
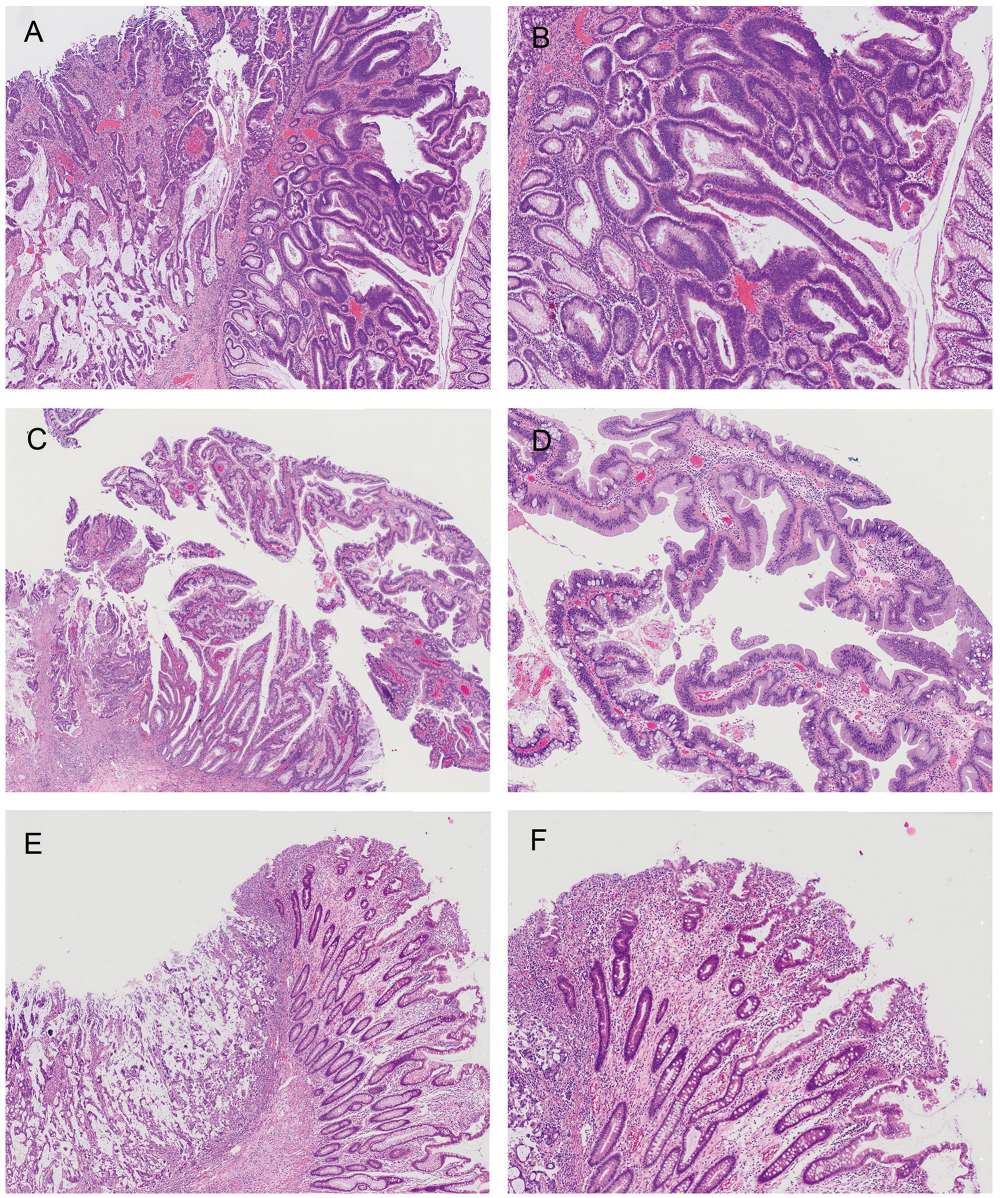
Lesions contiguous with the malignant tumor MAC without SAC morphology arising in a tubulovillous adenoma, showing a mixture of tubular and villous structures and dysplastic epithelium with elongated and hyperchromatic nuclei **(A & B)** MAC with SAC morphology arising in a traditional serrated adenoma showing ectopic crypts, crypt serration, and abundant eosinophilic cytoplasm **(C & D)** There were a few serrated glands around MACs with SAC morphology, but the size was not large enough to be diagnosed as a serrated polyp **(E & F)** Histological SAC features of the cancer were focally seen, and were not demonstrated in the figure.

Histological SAC features were observed in both mucinous and non-mucinous areas, but their distribution was not diffuse in most MACs with SAC morphology. Only two MACs with SAC morphology showed diffuse epithelial serration (>50% of the tumor volume). The most common contiguous precursor lesions were tubulovillous adenomas (Figure 2A–2B). Contiguous traditional serrated adenomas (Figure 2C–2D) were observed in 4 (12.5%) MACs with SAC morphology, but were not seen in any MACs without SAC morphology (*P*=0.019) (Supplementary Table 1). Three of these four tumors were located in the proximal colon. Contiguous sessile serrated adenomas were not observed; however, a few serrated glands around the malignant tumor (Figure 2E–2F) were noted in three (9.4%) MACs with SAC morphology.

### KRAS and BRAF mutations and CIMP status in colorectal MACs

Mutation analysis of *KRAS* codons 12 and 13 in exon 2 was performed successfully in 79 colorectal MACs; 34 (43%) of these had *KRAS* mutations. The most common KRAS mutation in our samples was G12D (16.5%, 13/79); other mutations included G13D (13.9%, 11/79), G12V (6.3%, 5/79), G12A (2.5%, 2/79), G12C (1.3%, 1/79), G12R (1.3%, 1/79), and A18D (1.3%, 1/79) (Supplementary Table 2).

Analysis of *BRAF* mutations in codon 600 of exon 15 was successfully performed in 77 colorectal MACs, and 12 (15.6%) exhibited *BRAF* mutations. The most common BRAF mutation in our series was V600E (13%, 10/77). Other BRAF mutations included D594G (1.3%, 1/77) and D594N (1.3%, 1/77) in two MACs without SAC morphology. BRAF mutation was associated with SAC morphology (*P*=0.042), poorer tumor differentiation (*P*=0.002), and CIMP-positive status (*P*=0.001) in colorectal MACs (Supplementary Table 3). No KRAS and BRAF mutations were identified in the same tumor.

CIMP analyses were performed successfully in 81 colorectal MACs. Fifteen percent (12/81) of these MACs had at least three aberrantly methylated genes and were CIMP-positive. CIMP-positive tumors were associated with SAC morphology (*P*=0.023), BRAF mutation (*P*=0.001), and wild-type KRAS (*P*=0.037) (Supplementary Table 4). BRAF mutation analysis was successfully performed in three of the four MACs with contiguous traditional serrated adenoma; all harbored BRAF mutations and were CIMP positive.

### MMR expression in colorectal MACs

Absence of MLH1 and MSH2 expression was observed in 15/88 (17%) and 5/88 (5.7%) colorectal MACs, respectively (Supplementary Table 5). All four MACs with contiguous traditional serrated adenoma were MMR proficient (pMMR), and three were located in the proximal colon.

### Association of clinicopathological and molecular characteristics with patient survival

**Figure 3:**
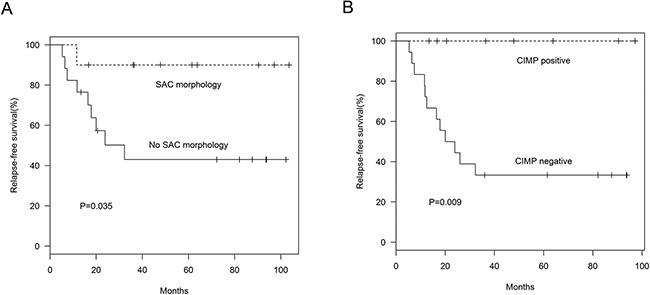
Relapse-free survival in AJCC stage III MAC patients SAC morphology **(A)** (*P*=0.035) and CIMP status **(B)** (*P*=0.009) were associated with relapse-free survival in AJCC stage III MAC patients.

Sixty patients with AJCC stage I–III MACs were included in relapse-free survival analyses. Seventeen patients had relapsed at the last follow-up (mean relapse-free survival after surgery, 14.51 months; range, 5.33–32.25 months). Mean follow-up duration for the 43 patients without relapse was 60.57 months (range, 9.24–106.82 months). In AJCC stage I–III MACs, tumor location, stage, and presence of SAC morphology or CIMP-positive status were associated with relapse-free survival (*P*<0.05 for all comparisons; Table 2). In AJCC stage III MACs, tumor location, pT status, SAC morphology (Figure 3A), CIMP status (Figure 3B), and presence of SAC morphology or CIMP-positive status were associated with relapse-free survival (*P*<0.05 for all comparisons; Table 2). In univariate analysis, AJCC stage III MACs without both SAC morphology and CIMP-positive status exhibited 10.567 times greater risk of cancer relapse than those having both characteristics or either one (*P*=0.025).

**Table 2:** Associations of MAC clinicopathologic and molecular characteristics with cancer relapse

Characteristic	Stage I to III			Stage III		
	No. of patients^a^	Relapse-free survival (%)	*P* Value	No. of patients^b^	Relapse-free survival (%)	*P*-value
Gender			0.605			0.255
Female	29	75.9		17	70.6	
Male	31	67.7		12	41.7	
Age			0.172			0.335
≤ 70 years	33	63.6		18	50	
> 70 years	27	81.5		11	72.7	
Location			0.001			< 0.001
Proximal colon	33	90.9		13	100	
Distal colon or rectum	27	48.1		16	25	
Differentiation			0.78			0.307
Well	10	80		5	80	
Moderate	43	69.8		19	47.4	
Poor	7	71.4		5	80	
pT status			0.225			0.043
pT1 or pT2 or pT3	51	74.5		26	65.4	
pT4	9	55.6		3	0	
pN status			0.091			0.578
pN0	31	83.9		-	-	
pN1	20	60		20	60	
pN2	9	55.6		9	55.6	
AJCC TNM stage			0.035	-	-	-
Stage I or II	31	83.9				
Stage III	29	58.6				
SAC morphology	(n=56)		0.144	(n=27)		0.035
Absent	34	67.6		17	47.1	
Present	22	86.4		10	90	
CIMP status	(n=56)		0.091	(n=26)		0.009
Negative	44	63.6		18	33.3	
Positive	12	91.7		8	100	
SAC morphology and CIMP-positive status	(n=54)		0.035	(n=25)		0.005
Presence of both or either one	26	88.5		12	91.7	
Absence of both	28	60.7		13	30.8	0.488
KRAS status	(n=55)		0.467	(n=28)		
Wild type	33	72.7		16	62.5	
Mutant	22	63.6		12	50	
BRAF status	(n=55)		0.643	(n=28)		0.214
Wild type	46	69.6		22	50	
Mutant	9	77.8		6	83.3	
MMR status			0.093			0.241
Proficient	44	65.9		21	52.4	
Defective	16	87.5		8	75	

AJCC, American Joint Committee on Cancer; CIMP, CpG island methylator phenotype; MAC, mucinous adenocarcinoma; MMR, DNA mismatch repair; HR, hazard ratio; SAC, serrated adenocarcinoma.

^a^ Values are calculated for 60 patients unless specified otherwise.

^b^ Values are calculated for 29 patients unless specified otherwise.

In multivariate analysis, AJCC stage I–III MACs without both SAC morphology and CIMP-positive status exhibited 3.955 times greater risk of cancer relapse than those having both characteristics or either one (*P*=0.035; Table 3) after adjusting for cancer stage. To avoid the effect of multicollinearity, we did not include the covariate “location” in this analysis. SAC morphology or CIMP status singly did not influence survival in the multivariate model (data not shown).

**Table 3:** Multivariable associations with cancer relapse in colorectal MAC patients

Characteristic	Stage I to III		Stage III	
	HR (95% CI)	*P* Value	HR (95% CI)	*P* Value
SAC morphology and CIMP-positive status				
Presence of both or either one	1.0 (reference)		1.0 (reference)	0.036
Absence of both	3.955 (1.101-14.214)	0.035	9.416 (1.157-76.642)	
AJCC TNM stage				
Stage I or II	1.0 (reference)		-	-
Stage III	3.719 (1.159-11.927)	0.027		
pT status				
pT1 or pT2 or pT3	-	-	1.0 (reference)	
pT4			2.033 (0.410-10.071)	0.385

AJCC, American Joint Committee on Cancer; HR, hazard ratio; MAC, mucinous adenocarcinoma; SAC, serrated adenocarcinoma.

## DISCUSSION

The histological criteria proposed by Makinen, *et al*. describe a colorectal cancer subtype with distinct serrated architecture associated with serrated polyps. Previous studies and ours confirmed that these criteria are feasible, with satisfactory interobserver reproducibility [18]. SAC incidence, and its association with various molecular features, had not previously been surveyed in colorectal MAC. We showed that MAC with SAC morphology tended to grow in the proximal colon with a higher frequency of “serrated pathway carcinoma” molecular features, including BRAF mutations and CIMP-positivity than MACs without SAC morphology. SAC morphology was noted in 38% of MACs in our study, a result higher than previously reported (9.1%) for SAC in colorectal cancer [18], which might explain the high frequency of BRAF mutations and CIMP-positive status in MACs.

Contiguous serrated precursor lesions have previously been observed in 52% of reported colorectal SACs [18]. We observed serrated precursor lesions in only four (13%) MACs with SAC morphology. These lesions were not observed in MACs without SAC morphology, which further suggests an association between serrated polyps and SACs. Notably, all serrated precursor lesions in our series were traditional serrated adenomas, which are the least frequently occurring serrated polyps. However, this was consistent with the findings of Bettington, *et al*., who observed that serrated morphology carcinomas most often arise from traditional serrated adenomas and tubulovillous adenomas with serrated features [13]. García-Solano, *et al*. postulated that precursor sessile serrated adenoma frequency is probably underestimated, because sessile serrated adenomas can undergo complete histological dysplasia and high-grade transformation, leading to a traditional serrated adenoma or in situ adenocarcinoma appearance [18, 27].

In contrast with previous conventional CRC findings (most are non-MAC) [18, 19], patients in our study who had colorectal MACs with SAC morphology exhibited improved relapse-free survival, and this was significant in AJCC stage III MAC patients. SAC morphology was associated with proximal cancer location and CIMP-positive status, which were favorable prognostic factors in our analyses.

Prior studies of CIMP status for predicting CRC patient prognosis have been controversial [28]. Discrepancies between studies might be explained by differences in the study populations or in the methodologies and panels used to determine CIMP status. The CIMP panel we used was identified by Weisenberger and colleagues, and was validated in CRC in a large, population-based cohort [29]. CIMP panels used in many previous studies were modified from the Weisenberger panel, with new markers added. In previous studies using the Weisenberger panel, CIMP-positive status suggested good patient outcome in KRAS wild-type or MSI CRC [28, 30], but a poor prognosis in rectal cancer or CRC with chromosomal instability [30, 31].

Our study had several limitations. First, we did not include non-MACs for comparison, as our findings on the prognostic implications of SAC morphology contradicted results from studies of conventional, mostly non-MAC, CRC [18, 19]. Second, to avoid the effect of neoadjuvant therapy in histological analyses, we excluded low rectal cancer cases, which had often undergone neoadjuvant concurrent chemoradiotherapy. Third, PMS2 and MSH6 were not analyzed via IHC. However, as cases with isolated loss of PMS2 or MSH6 expression were uncommon [32], it is unlikely that this exclusion affected our main findings.

In conclusion, our results showed that two MAC groups with distinct clinicopathological and molecular features could be identified using Makinen's criteria, and suggested a favorable prognostic role for the serrated neoplastic pathway in colorectal MAC. The relatively high SAC incidence in MACs might explain the high frequency of BRAF mutations and CIMP-positive status in MACs. A more complete understanding of the prognostic role of the serrated neoplastic pathway in colorectal MAC and conventional CRC (mostly non-MAC) will require further research.

## MATERIALS AND METHODS

### Patients and materials

The Institutional Review Board of the National Cheng Kung University Hospital approved this study. We identified 88 surgically resected colorectal MACs without neoadjuvant therapy by searching our electronic pathology database for records dated between 2007 and 2014. Signet-ring cell carcinomas were excluded. Tumor status was recorded according to the AJCC cancer-staging manual (7^th^ ed.). Adjuvant chemotherapy with 5-FU was administered to patients with AJCC stage II–IV cancers following standard schedules and doses. Histology was reviewed for all cases, and diagnoses were confirmed. Six patients had one or two synchronous MACs or non-MACs, and in these cases we analyzed the largest MAC. No patient had a family history of Lynch syndrome. Proximal cancers were defined as those in the cecum, ascending, and transverse colon.

### Histological evaluation of SAC morphology

Three pathologists with >10 years of experience (Lee C. T., Tsai H. W., and Ho C. L.) reviewed the sections. MAC was diagnosed when more than half of the tumor volume was composed of extracellular mucin-containing tumor cells [1, 2]. “SAC morphology” was defined according to established Makinen's criteria [13, 15, 17], including epithelial serrations, clear or eosinophilic cytoplasm, abundant cytoplasm, vesicular nuclei, distinct nucleoli, scarceness (<10%) of necrosis, mucin production, and cell balls or papillary rods in the mucin. Histologic evaluation details are provided in the Supplementary Methods.

“SAC morphology” for a well or moderately differentiated MAC was considered positive when the cancer met at least six of the first seven features listed above [13, 18]. We omitted the “epithelial serration” criterion for poorly differentiated MACs without glandular architecture, and those that met at least five of the first seven features were categorized as having SAC morphology. Differences in opinion for SAC morphology diagnosis were resolved by re-evaluating the sections to reach a consensus.

Any lesions contiguous with the cancer were also evaluated. Conventional (tubular, tubulovillous, and villous) adenoma, sessile serrated adenoma, and traditional serrated adenoma were classified according to WHO classifications [1].

### DNA extraction and BRAF and KRAS mutation analysis

Ten-micrometer-thick sections were cut from paraffin-embedded tumor samples, placed on slides, and the tumor area was scraped off. DNA was extracted using standard phenol chloroform methods. Mutations covering *KRAS* codons 12 and 13, and *BRAF* codon 600 were assessed with direct sequencing of PCR-amplified DNA (Supplementary Methods).

### CIMP analysis

CIMP status was assessed using the Weisenberger panel (CACNA1G, IGF2, NEUROG1, RUNX3, and SOCS1) with an automated real-time, PCR-based MethyLight system as previously described (Supplementary Methods) [33]. All primer sequences were published previously [33]. Tumor samples were categorized as CIMP positive if methylation was detected in ≥3/5 genes, and CIMP negative if methylation was detected in ≤2/5 genes.

### Immunohistochemistry (IHC)

IHC for MLH1 and MSH2 was performed using an avidin-biotin complex-peroxidase procedure with an automated stainer (Supplementary Methods). A pathologist (Lee C. T.) assessed all IHC stains. MLH1 or MSH2 expression was defined as abnormal when nuclear staining of tumor cells was absent despite positive staining in surrounding stromal cells. Defective MMR (dMMR) was defined as loss of MLH1 or MSH2 expression in tumor cells. Proficient MMR (pMMR) was defined by the presence of MLH1 and MSH2 expression in tumor cells [34].

### Statistical analysis

Relationships between categorical characteristics were analyzed via χ^2^ test or Fisher's exact test. κ values were calculated to assess observer agreement for SAC morphology diagnosis. κ<0.2 indicated poor agreement, 0.21–0.40 was fair, 0.41–0.60 was moderate, 0.61–0.80 was good and >0.8 was very good [35].

Relapse-free survival was calculated from the date of surgery. Events were defined as any disease recurrence shown histologically or via imaging. Survival analyses included only patients with AJCC stage I–III colorectal MACs. Patients who were followed-up for <3 years were excluded unless an event occurred or the patient had died. Patients who died within one month of surgery and patients who had more than one cancer were also excluded from survival analyses. The significance of various covariates was assessed using univariate analysis with the log-rank test. Survival curves were calculated using the Kaplan-Meier method. A multivariate model was constructed through stepwise selection with *P* set at 0.05 for a characteristic to be included or excluded from the model. All tests were 2-sided, and *P*<0.05 was considered statistically significant.

## SUPPLEMENTARY MATERIALS TABLES


