# Supplementary material for: Serrated adenocarcinoma morphology in colorectal mucinous adenocarcinoma is associated with improved patient survival
Source: Oncotarget. 2017 Apr 4;8(21):35165–75. doi: 10.18632/oncotarget.16815 (PMC5471043; doi:10.18632/oncotarget.16815)
Supplement: Supplementary file 1 [file oncotarget-08-35165-s001.pdf]

# Serrated adenocarcinoma morphology in colorectal mucinous adenocarcinoma is associated with improved patient survival

## SUPPLEMENTARY METHODS

### Histological evaluation of SAC morphology

Epithelial serration was characterized by epithelial tufts containing epithelium with or without basement membrane material. Papillary projections with fibrovascular cores and serrate-like structures resulting from tumor cell necrosis were excluded. The cut-off value for "epithelial serration," "clear or eosinophilic cytoplasm," or "abundant cytoplasm" was set at 10%. Histological features were considered positive if they occurred in more than 10% of the tumor volume. "Distinct nucleoli" were defined as nucleoli that were easily recognizable under a 10x microscope objective. "Tumor necrosis" was defined as glandular lumina filled with inspissated material containing nuclear and cellular debris as described previously.[1]

### DNA extraction and KRAS and BRAF mutation analysis

Ten-micrometer-thick sections were cut from paraffin-embedded tumor samples. The sections were placed on glass slides, and the tumor area was scraped off with a surgical blade. DNA was extracted with standard phenol chloroform methods. Mutations covering KRAS codons 12 and 13 and BRAF codon 600 were assessed with direct sequencing of polymerase chain reaction (PCR)-amplified DNA. The following primers were used: TGC TTG CTC TGA TAG GAA AAT G (forward) and AGC ATC TCA GGG CCA AAA AT (reverse) for BRAF codon 600, CTG GTG GAG TAT TTG ATA GTG (forward) and TGG TCC TGC ACC AGT AAT ATG C (reverse) for KRAS codons 12 and 13. The same primers were used for amplification and sequencing.

The PCR conditions consisted of initial denaturing at 95°C for 6 minutes; then 35 cycles of 95°C for 30 seconds, 60°C for 1 minute, 72°C for 1 minute; and 72°C for 10 minutes. PCR products (5 µL) were analyzed by electrophoresis on a 2% agarose gel to confirm amplicon size, and 5 µL PCR product was purified with EXOSAP (Affymetrix USB, Cleveland, Ohio). Purified DNA was used as a template with 0.8 µM Sanger sequencing primers for a sequencing reaction with the BigDye Terminator v1.1 Cycle Sequencing Kit (Applied Biosystems, Forster City, CA). The mixtures were run through the following program in the thermocycler: 1 minute for initial denaturation of the DNA at 96°C followed by 25 cycles of a 10 second denaturation at 96°C, annealing of the primer at 50°C for 5 seconds, and an extension step at 60°C for 4 minutes. Capillary electrophoresis was performed on a 3500 Genetic Analyzer after completion of the sequencing program. The sequencing results were aligned with the KRAS and BRAF reference sequences with NCBI Blast. The accession numbers for the KRAS and BRAF reference sequences are NG_007524.1 and NG_007572.1. All of the sequences were verified in the forward and reverse directions.

### CIMP analysis

Ten-micrometer-thick sections were cut from paraffin-embedded tumor samples. The sections were placed on glass slides, and the tumor area was scraped off with a surgical blade. DNA extraction was performed using the QIAamp tissue kit (Qiagen, Valencia, CA) in accordance with the manufacturer's instructions. CIMP status was evaluated by treating tumor DNA with an EpiTect Fast DNA Bisulfite kit (Qiagen, Valencia, CA) and subsequently analyzed with an automated real-time, PCR-based MethyLight system that quantitatively measures genome-specific DNA methylation levels, as compared with a methylated reference sample (M.SssI-treated DNA), to calculate the methylated reference value for each sample and gene region as previously reported.[2] ALU (in Alu repeats) was used to normalize the input quantity of bisulfite-treated genomic DNA. PCR primers, TaqMan probes, and reaction components for real-time PCR were purchased from Applied Biosystems (Foster, CA) to amplify methylated CpG sites in the promoter regions of an established 5-gene marker panel (CACNA1G, IGF2, NEUROG1, RUNX3, and SOCS1).[2] All of the primer and probe sequences were published previously.[2] PCR conditions were initial denaturation at 95°C for 10 minutes followed by 40 cycles of 95°C for 15 seconds and 60°C for 1 minute. Tumor samples with ALU C(t) > 25 were considered unsuitable and excluded from the CIMP data analyses. The percentage of methylated reference (PMR) at each locus was calculated by dividing the ratio of GENE/ALU in a sample by the ratio of GENE/ALU in SssI-treated human genomic DNA (presumably fully methylated) and multiplying this value by 100. Positive methylation at each locus was defined as PMR > 10 as previously described.[2] Tumor samples were categorized as CIMP positive if methylation was detected in $\geq$ 3 of five genes, and CIMP negative if positive methylation was detected in $\leq$ 2 of five genes.

### Immunohistochemistry (IHC)

IHC for MLH1 and MSH2 was performed using a standard avidin-biotin complex-peroxidase procedure with an automated stainer (Leica, Melbourne, Australia). Briefly, 4-µm tissue sections were obtained from a representative formalin-fixed, paraffin-embedded tissue block of each tumor. Sections were deparaffinized and rehydrated, and heat-induced epitope retrieval was performed using Bond Epitope Retrieval Solution 2 (Leica Biosystems, Newcastle, UK) at 100°C for 30 minutes. The slides were incubated with primary antibodies for MLH1 (diluted 1:50; Novocastra, Newcastle, UK) or MSH2 (diluted 1:75; Zeta Corporation, Arcadia, CA). MLH1 and MSH2 proteins were detected and visualized using a Bond Polymer Refine Detection kit (Leica Biosystems, Newcastle, UK). The slides were incubated for 8 minutes, counterstained with hematoxylin and coverslipped. Positive and negative controls were included in all of the runs. Negative controls omitted the primary antibodies, and positive controls were tissues known to express the proteins of interest.

## REFERENCES


1. Garcia-Solano J, Perez-Guillermo M, Conesa-Zamora P et al. Clinicopathologic study of 85 colorectal serrated adenocarcinomas: further insights into the full recognition of a new subset of colorectal carcinoma. Hum Pathol 2010; 41: 1359-68.
2. Weisenberger DJ, Siegmund KD, Campan M et al. CpG island methylator phenotype underlies sporadic microsatellite instability and is tightly associated with BRAF mutation in colorectal cancer. Nat Genet 2006; 38: 787-93.

**Supplementary Table 1: Histological SAC features and contiguous lesions observed in 84 colorectal MACs**

| Histologic features | MAC without SAC morphology (n=52)[a] | MAC with SAC morphology (n=32)[b] | |
|---|---|---|---|
| **Histologic criteria of SAC** | | | |
| 1. Epithelial serration (n=79)[c] | 17 (34.7) | 23 (76.7) | |
| 2. Eosinophilic or clear cytoplasm | 39 (75) | 32 (100) | |
| 3. Abundant cytoplasm | 4 (7.7) | 21 (65.6) | |
| 4. Vesicular nuclei | 30 (57.7) | 32 (100) | |
| 5. Distinct nucleoli | 26 (50) | 32 (100) | |
| 6. Scarceness (< 10%) of necrosis | 43 (82.7) | 30 (93.8) | |
| 7. Intracellular and extracellular mucin | 52 (100) | 32 (100) | |
| 8. Cell balls and papillary rods in mucin | 40 (76.9) | 29 (90.6) | |
| **Contiguous lesions** | | | ***P* value** |
| Tubulovillous adenoma | 13 (25) | 6 (18.8) | 0.506 |
| Tubular adenoma | 3 (5.8) | 0 | 0.284 |
| Traditional serrated adenoma | 0 | 4 (12.5) | 0.019 |
| Serrated glands | 0 | 3 (9.4) | 0.052 |

Abbreviations: MAC, mucinous adenocarcinoma; SAC, serrated adenocarcinoma.
[a] Values are expressed for 52 MACs without SAC morphology unless specified otherwise, and are presented as number and percentage of patients.
[b] Values are expressed for 32 MACs with SAC morphology unless specified otherwise, and are presented as number and percentage of patients.
[c] Five poorly differentiated MACs were excluded.

**Supplementary Table 2: Clinical, pathologic, and molecular characteristics in colorectal MACs with and without KRAS mutation**

| Characteristic[a] | KRAS wild-type (n=45)[b] | KRAS mutation (n=34)[b] | *P* value |
|---|---|---|---|
| Gender | | | 0.817 |
| Male | 25 (55.6) | 18 (52.9) | |
| Female | 20 (44.4) | 16 (47.1) | |
| Age | | | 0.972 |
| ≤ 70 y/o | 24 (53.3) | 18 (52.9) | |
| > 70 y/o | 21 (46.7) | 16 (47.1) | |
| Location | | | 0.074 |
| Proximal colon | 25 (55.6) | 12 (35.3) | |
| Distal colon or rectum | 20 (44.4) | 22 (64.7) | |
| Differentiation | | | |
| Well[A] | 4 (8.9) | 8 (23.5) | 0.206 (A vs. B) |
| Moderate[B] | 31 (68.9) | 24 (70.6) | 0.036 (A vs. C) |
| Poor[C] | 10 (22.2) | 2 (5.9) | 0.108 (B vs. C) |
| pT status | | | |
| pT1 or pT2[D] | 4 (8.9) | 0 (0) | 0.287 (D vs. E) |
| pT3[E] | 34 (75.6) | 23 (67.6) | 0.090 (D vs. F) |
| pT4[F] | 7 (15.6) | 11 (32.4) | 0.123 (E vs. F) |
| pN status | | | |
| pN0[G] | 22 (48.9) | 12 (35.3) | 0.235 (G vs. H) |
| pN1[H] | 15 (33.3) | 15 (44.1) | 0.451 (G vs. I) |
| pN2[I] | 8 (17.8) | 7 (20.6) | 0.833 (H vs. I) |
| Distant metastasis | 6 (13.3) | 7 (20.6) | 0.389 |
| AJCC TNM stage | | | |
| Stage I or II[J] | 21 (46.7) | 12 (35.3) | 0.453 (J vs. K) |
| Stage III[K] | 18 (40.0) | 15 (44.1) | 0.278 (J vs. L) |
| Stage IV[L] | 6 (13.3) | 7 (20.6) | 0.608 (K vs. L) |
| SAC morphology (n = 75) | 17 (39.5) | 13 (40.6) | 0.924 |
| CIMP positive (n = 73) | 9 (21.4) | 1 (3.2) | 0.037 |
| dMMR | 15 (33.1) | 4 (11.8) | 0.034 |

Abbreviations: MAC, mucinous adenocarcinoma; SAC, serrated adenocarcinoma; AJCC, American Joint Committee on Cancer; CIMP, CpG island methylator phenotype; dMMR, defective mismatch repair protein.

[a] Values are expressed for 79 patients unless specified otherwise.

[b] Values are presented as number and percentage of patients.

**Supplementary Table 3: Clinical, pathologic, and molecular characteristics in colorectal MACs with and without BRAF mutation**

| Characteristic[a] | BRAF wild-type (n=65)[b] | BRAF mutation (n=12)[b] | *P* value |
|---|---|---|---|
| Gender | | | 0.329 |
| Male | 37 (56.9) | 5 (41.7) | |
| Female | 28 (43.1) | 7 (58.3) | |
| Age | | | 0.282 |
| ≤ 70 y/o | 38 (58.5) | 5 (41.7) | |
| > 70 y/o | 27 (41.5) | 7 (58.3) | |
| Location | | | 0.208 |
| Proximal colon | 28 (43.1) | 8 (66.7) | |
| Distal colon or rectum | 37 (56.9) | 4 (33.3) | |
| Differentiation | | | |
| Well[A] | 10 (15.4) | 3 (25.0) | 0.122 (A vs. B) |
| Moderate[B] | 51 (78.5) | 4 (33.3) | 0.187 (A vs. C) |
| Poor[C] | 4 (6.2) | 5 (41.7) | 0.002 (B vs. C) |
| pT status | | | |
| pT1 or pT2[D] | 3 (4.6) | 0 (0) | 1.000 (D vs. E) |
| pT3[E] | 47 (72.3) | 10 (83.3) | 1.000 (D vs. F) |
| pT4[F] | 15 (23.1) | 2 (16.7) | 0.721 (E vs. F) |
| pN status | | | |
| pN0[G] | 28 (43.1) | 5 (41.7) | 0.869 (G vs. H) |
| pN1[H] | 25 (38.5) | 5 (41.7) | 1.000 (G vs. I) |
| pN2[I] | 12 (18.5) | 2 (16.7) | 1.000 (H vs. I) |
| Distant metastasis | 10 (15.4) | 2 (16.7) | 1 |
| AJCC TNM stage | | | |
| Stage I or II[J] | 28 (43.1) | 4 (33.3) | 0.733 (J vs. K) |
| Stage III[K] | 27 (41.5) | 6 (50.0) | 0.658 (J vs. L) |
| Stage IV[L] | 10 (15.4) | 2 (16.7) | 1.000 (K vs. L) |
| SAC morphology (n = 73) | 22 (35.5) | 8 (72.7) | 0.042 |
| CIMP positive (n = 71) | 4 (6.7) | 6 (54.5) | 0.001 |
| dMMR | 14 (21.5) | 5 (41.7) | 0.137 |

Abbreviations: MAC, mucinous adenocarcinoma; SAC, serrated adenocarcinoma; AJCC, American Joint Committee on Cancer; CIMP, CpG island methylator phenotype; dMMR, defective mismatch repair protein.
[a] Values are expressed for 77 patients unless specified otherwise.
[b] Values are presented as number and percentage of patients.

**Supplementary Table 4: CIMP status and its correlation with clinical, pathologic, and molecular characteristics in colorectal MACs**

| Characteristic[a] | CIMP negative (n=69)[b] | CIMP positive (n=12)[b] | *P* value |
|---|---|---|---|
| Gender | | | 0.542 |
| Male | 41 (59.4) | 6 (50.0) | |
| Female | 28 (40.6) | 6 (50.0) | |
| Age | | | 0.675 |
| ≤ 70 y/o | 39 (56.5) | 6 (50.0) | |
| > 70 y/o | 30 (43.5) | 6 (50.0) | |
| Location | | | 0.067 |
| Proximal colon | 31 (44.9) | 9 (75.0) | |
| Distal colon or rectum | 38 (55.1) | 3 (25.0) | |
| Differentiation | | | |
| Well[A] | 11 (15.9) | 3 (25.0) | 0.407 (A vs. B) |
| Moderate[B] | 49 (71.0) | 7 (58.3) | 1.000 (A vs. C) |
| Poor[C] | 9 (13.0) | 2 (16.7) | 0.635 (B vs. C) |
| pT status | | | |
| pT1 or pT2[D] | 8 (11.6) | 0 (0) | 0.330 (D vs. E) |
| pT3[E] | 43 (62.3) | 11 (91.7) | 1.000 (D vs. F) |
| pT4[F] | 18 (26.1) | 1 (8.3) | 0.166 (E vs. F) |
| pN status | | | |
| pN0[G] | 36 (52.2) | 4 (33.3) | 0.288 (G vs. H) |
| pN1[H] | 20 (29.0) | 5 (41.7) | 0.395 (G vs. I) |
| pN2[I] | 13 (18.8) | 3 (25.0) | 1.000 (H vs. I) |
| Distant metastasis | 11 (15.9) | 0 (0) | 0.203 |
| AJCC TNM stage | | | |
| Stage I or II[J] | 36 (52.2) | 4 (33.3) | 0.108 (J vs. K) |
| Stage III[K] | 22 (31.9) | 8 (66.7) | 0.565 (J vs. L) |
| Stage IV[L] | 11 (15.9) | 0 (0) | 0.083 (K vs. L) |
| SAC morphology (n = 77) | 23 (34.8) | 8 (72.7) | 0.023 |
| KRAS mutation (n = 73) | 30 (47.6) | 1 (10) | 0.037 |
| BRAF mutation (n = 71) | 5 (8.2) | 6 (60) | 0.001 |
| dMMR | 14 (20.3) | 5 (41.7) | 0.107 |

Abbreviations: MAC, mucinous adenocarcinoma; SAC, serrated adenocarcinoma; AJCC, American Joint Committee on Cancer; CIMP, CpG island methylator phenotype; dMMR, defective mismatch repair protein.

[a] Values are expressed for 81 patients unless specified otherwise.

[b] Values are presented as number and percentage of patients.

**Supplementary Table 5: Expression of mismatch repair proteins and its correlation with clinical, pathologic, and molecular characteristics in colorectal MACs**

| Characteristic[a] | pMMR (n=68)[b] | dMMR (n=20)[b] | *P* value |
|---|---|---|---|
| Gender | | | 0.484 |
| Male | 40 (58.8) | 10 (50.0) | |
| Female | 28 (41.2) | 10 (50.0) | |
| Age | | | 0.114 |
| ≤ 70 y/o | 34 (50.0) | 14 (70.0) | |
| > 70 y/o | 34 (50.0) | 6 (30.0) | |
| Location | | | 0.002 |
| Proximal colon | 27 (39.7) | 16 (80.0) | |
| Distal colon or rectum | 41 (60.3) | 4 (20.0) | |
| Differentiation | | | |
| Well[A] | 11 (16.2) | 3 (15.0) | 1.000 (A vs. B) |
| Moderate[B] | 49 (72.1) | 13 (65.0) | 0.665 (A vs. C) |
| Poor[C] | 8 (11.8) | 4 (20.0) | 0.454 (B vs. C) |
| pT status | | | |
| pT1 or pT2[D] | 6 (8.8) | 2 (10.0) | 1.000 (D vs. E) |
| pT3[E] | 44 (64.7) | 15 (75.0) | 0.597 (D vs. F) |
| pT4[F] | 18 (26.5) | 3 (15.0) | 0.373 (E vs. F) |
| pN status | | | |
| pN0[G] | 32 (47.1) | 10 (50.0) | 0.557 (G vs. H) |
| pN1[H] | 21 (30.9) | 9 (45.0) | 0.259 (G vs. I) |
| pN2[I] | 15 (22.1) | 1 (5.0) | 0.130 (H vs. I) |
| Distant metastasis | 13 (19.1) | 0 (0) | 0.034 |
| AJCC TNM stage | | | |
| Stage I or II[J] | 31 (45.6) | 10 (50.0) | 0.624 (J vs. K) |
| Stage III[K] | 24 (35.3) | 10 (50.0) | 0.095 (J vs. L) |
| Stage IV[L] | 13 (19.1) | 0 (0) | 0.043 (K vs. L) |
| SAC morphology (n = 84) | 25 (38.5) | 7 (36.8) | 0.898 |
| KRAS mutation (n = 79) | 30 (50.0) | 4 (21.1) | 0.034 |
| BRAF mutation (n = 77) | 7 (12.1) | 5 (26.3) | 0.137 |
| CIMP positive (n = 81) | 7 (11.3) | 5 (26.3) | 0.107 |

Abbreviations: MAC, mucinous adenocarcinoma; SAC, serrated adenocarcinoma; AJCC, American Joint Committee on Cancer; CIMP, CpG island methylator phenotype; pMMR, proficient mismatch repair protein; dMMR, defective mismatch repair protein.

[a] Values are expressed for 88 patients unless specified otherwise.

[b] Values are presented as number and percentage of patients.
